# More Than Just an Abscess: Ultrasound-Assisted Diagnosis of Ventriculoperitoneal Shunt Infection

**DOI:** 10.5811/westjem.2015.8.27984

**Published:** 2015-12-08

**Authors:** Erik A. Berg, Saman Kashani, Tarina L. Kang

**Affiliations:** LAC+USC Medical Center, Department of Emergency Medicine, Los Angeles, California

A 60-year-old female with a history of ventriculoperitoneal shunt (VPS) placement three years prior presented with a painful abdominal wall mass. The patient denied fevers, nausea, vomiting, headaches, or dizziness. Physical exam revealed an afebrile, well-appearing female with a raised, erythematous, fluctuant mass on the right lower abdominal wall. She had no abdominal tenderness otherwise. Labs were unremarkable. A bedside ultrasound revealed a complex fluid collection over the area of fluctuance that tracked along the course of the VPS tubing into the abdomen. Plan for incision and drainage was deferred. Neurosurgery was consulted. The neurosurgeon attempted to tap the shunt but encountered very high resistance. The patient was admitted for intravenous antibiotics for VPS infection and malfunction.

VPSs are neurosurgically implanted devices used to treat hydrocephalus by shunting cerebral spinal fluid from the lateral ventricles of the brain into the peritoneum. Shunt infections, including meningitis, ventriculitis, and peritonitis, occur in 2–17% of VPS cases.^1^–^3^ Clinicians should maintain a high index of suspicion for VP shunt complications in patients who present with typical symptoms suggestive of increased intracranial pressure. In this case, a less obvious complication such as an abscess in an atypical location lowered the practitioner’s threshold for bedside imaging and further investigation.

## Figures and Tables

**Video:** Circumferential fluid collection surrounding ventriculoperitoneal shunt (white arrow).
